# Risk assessment and quantitative measurement along with monitoring of *Legionella* in hospital water sources

**DOI:** 10.1016/j.nmni.2021.100948

**Published:** 2021-12-24

**Authors:** S. Bavari, S. Mirkalantari, F. Masjedian Jazi, D. Darban-Sarokhalil, B. Golnari Marani

**Affiliations:** 1) Microbiology Department, School of Medicine, Iran University of Medical Sciences, Tehran, Iran; 2) Cellular and Molecular Research Center, Iran University of Medical Sciences, Tehran, Iran

**Keywords:** HeLa cell, hospital water sources, invasion, *Legionella*, PCR, quantitative PCR

## Abstract

*Legionella* spp. as a causative agent of Legionnaires' disease (LD) and an opportunistic pathogen creates a public health problem. Isolation and quantification of this bacteria from clinic water sources are essential for hazard appraisal and sickness avoidance. This study aimed at risk assessment and quantitative measurement along with *Legionella* monitoring in educational hospital water sources in Tehran, Iran. A cross-sectional study was carried out in 1 year. The conventional culture method was used in this study to isolate *Legionella* from water samples. The polymerase chain reaction (PCR) technique was used to confirm the identity of the isolates and ensure that they were all *Legionella*. Quantitative PCR (qPCR) was used to determine the count of bacteria, and HeLa cell culture was used to determine the invasion of isolates. A total of 100 water samples were collected and inoculated on GVPC (glycine, vancomycin, polymyxin, and cycloheximide) agar; 12 (12%) and 42 (42%) cases were culture and PCR positive, respectively. Percentage of *Legionella* presence in PCR-positive samples by the qPCR method in <10^3^ GU/L, in about 10^3^ and lower than 10^4^ GU/L, and in 10^4^ GU/L was 40.47 (17 cases), 4.76% (two cases), and 54.76% (23 cases), respectively. Invasion analysis revealed that five and four isolates had invaded HeLa cells more than twice and equally, respectively, and the others had a lower invasion than the reference strain. The findings revealed that the spread of LD in hospitals was linked to the water system. Given the importance of nosocomial infections in the medical community, establishing a hospital water monitoring system is the most effective way to control these infections, particularly *Legionella* infections.

## Introduction

*Legionella* is a bacteria found in natural places, artificial water resources, and ventilation systems [[1], [2], [3]]. This bacteria causes sporadic pneumonia and acquired epidemics from the community (CAP: community-acquired pneumonia; 1–3%) or the hospital (NAP: nosocomial acquired pneumonia; 30%) in healthy subjects with immunodeficiency [4,5]. Large water systems in large buildings, such as hospitals, are often infected by *Legionella* and therefore cause potential danger to patients [6]. The pneumonia rate in the hospital due to *Legionella* was reported to be 0 to 47% [7]. Legionellosis in the hospital is underestimated for various reasons, including lack of clinical awareness or inappropriate diagnosis [[7], [8], [9]]. *Legionella* contamination in the hospital water supply sources is associated with the outbreak of nosocomial Legionnaires' disease (LD) [10]. Therefore, testing of hospital water systems is essential to risk assessment of nosocomial infections of *Legionella*.

To measure the count of *Legionella* in water samples, quantitative polymerase chain reaction (qPCR) is practical. This method is a new modification of the PCR method [11] and an alternative method for rapid enumeration of *Legionella* spp. from environmental samples [10]. It simultaneously amplifies and quantifies a target DNA sequence [12], giving the genome units (GU) per litre [13]. Although the cultivation method for the isolation of *Legionella* is a gold standard and approved by ISO and many other national standards for determining water quality, over the past few years, 16S rRNA gene molecular techniques have been developed in addition to other genetic markers [14,15]. The *Legionella* density theoretically influences the risk of Legionellosis in the water sources [16,17]. Previous studies reported that densities above 10^4^ to 10^5^ CFU/litre (10^4^–10^10^ *Legionella* CFU/L) [18] represent a potential increased threat to human health [19,20]. Conventional culture is generally used to detect and count *Legionella* in water samples, but it can take up to 10 days to obtain a firm result; besides, the culture sensitivity is low (10–30%) [21,22], especially when samples contain microorganisms that inhibit *Legionella* growth. Also, *Legionella* cells that are viable but non-culturable are not detected by conventional culture [23,24], and they are yet potentially pathogenic [25].

The HeLa cell culture, which was introduced as a model for the invasion and biology of *Legionella pneumophila* (*L. pneumophila*), was used to measure the invasion rate in our study [[26], [27], [28], [29]]and showed that *Legionella* virulent strains virtually invade the HeLa cells and non-virulent strains have less ability in the invasion. Based on the above, this study aimed to risk assessment and quantitative measurement and monitor *Legionella* in hospital water sources at the Iran University of Medical Sciences (IUMS, Tehran, Iran).

## Material and methods

### Sample collection

In a cross-sectional study, 100 water samples were collected from nine different educational hospital water sources of the IUMS (Tehran) over 1 year (2019–2020). All samples were temperature, pH, and residual chlorine assessed and then transmitted to the microbiology laboratory in less than 2 hours using a clean, sterile bottle containing disinfection neutralizing agents such as sodium thiosulphate. Centrifugation at 2500×g for 10 minutes concentrated the samples. The sediment was resuspended in 5 mL of the same water after the supernatant was removed.

### Sample preparation and culture

Inoculation of samples was done after acid (HCL-KCL buffer, pH 2.2) treatment [30]. One hundred microliters (0.1 mL = 100μL) of the concentrated sample were directly plated on a selective GVPC medium (glycine, vancomycin, polymyxin, and cycloheximide) agar that recommended by ISO 11731:2017 for the isolation of *Legionella*. Plates were incubated in a humid environment under a microaerophilic in a candle jar or under a 2.5% CO_2_ atmosphere at 35°C (CDC, 2005) for about 2 weeks and checked daily. The colonies were subcultured on GVPC agar and the ordinary media such as Blood agar and buffered charcoal yeast extract (BCYE) media without L-Cys. Gram staining was performed on isolates that did not grow on Blood agar. With specific primers, PCR was used to examine isolates that were suspected to be *Legionella*.

### DNA extraction

Water samples that had been concentrated were stored at −20°C. One millilitre of each stored sample was centrifuged for 5 minutes at 14 000 rpm. The DNA was extracted from the sediment. A commercial kit (Favorgen Biotech, Taiwan) was used to extract DNA from water samples, and the manufacturer's instructions were followed. A spectrophotometer was used to check the quality of each sample of extracted DNA (Thermo Scientific). The extracted DNA was stored at −20°C until performing molecular methods, including PCR and qPCR [31].

### PCR assay

To perform genus-specific PCR, for *Legionella* molecular identification, following primers of 16S rRNA gene, 5 μL of template DNA in 25 μL reaction mixture composed of 1 nM (1 μL) forward (AGG GTT GAT AGG TTA AGA GC) and 1 nM (1 μL) reverse (CCA ACA GCT AGT TGA CAT CG) primers (Table 1), 8 μL master mix (Amplicon) and 10 μL the sterile deionized water. The microtubes were transferred to a thermal cycler (PeqLab Biotechnology, Erlangen, Germany). For amplification, an initial denaturing step of 5 minutes at 95°C, 30 cycles of 1 minute at 94°C, 1 minute at 54°C, 45 seconds at 72°C, and 5 minutes as a final extension at 72°C were performed. The PCR products were electrophoresed in a 1.5% agarose gel containing a 1 μL safe stain. The PCR product size was 386-bp. DNA of *L. pneumophila* (ATCC 33152) was used in all PCR runs as a positive control. A reaction without any DNA runs as a negative control.

**Table 1:** Primers used for 16S rRNA gene PCR

Target gene	Oligonucleotide sequence 5′-3′	Product size (bp)	Reference
16S rRNA	F: AGG GTT GAT AGG TTA AGA GC	386	This study
	R: CCA ACA GCT AGT TGA CAT CG		

### qPCR assay conditions

The real-time PCR assay was performed using QIAGEN's real-time PCR cycler (Applied Rotor gene, Germany) with a total reaction volume of 10 μL. Each reaction mix contained 5 μL sterile deionized water, 0.25 nM each of the primer (0.5 nM F and R), 4 μL Real Q plus Master Mix Green (Amplicon; 2×), and 0.5 μL template DNA. For *Legionella* spp., primers were used to amplify 212bp fragments. In this study, primer sets 16S rRNA-F (5′-CAG ATA ATA CTG GTT GAC TC-3′) and 16S rRNA-R (5′-TTC ATA TAA CCA ACA GCT AG-3′) were used for the detection of *Legionella* spp. count. Cycling parameters for real-time PCR analysis included predenaturation for 15 minutes at 95°C, followed by 45 cycles of 15 seconds denaturation at 95°C, annealing at 60°C for the 20 seconds, and a final extension at 72°C for 20 seconds. SYBR green real-time PCR assays performed melt curve analysis to verify specificity by increasing the temperature from 65 to 95°C at a rate of 0.1 °C/s. For each assay, the threshold cycle (Ct) value, defined as the PCR cycle at which the fluorescence signal increases above the background threshold, determines the quantification of each DNA product. The standard was prepared 6-fold dilution series from 10^5^ to 10^0^ were used as a template from *L. pneumophila* (ATCC 33152). Negative DNA control was distilled water that was replaced with template DNA. Controls and samples were analysed in duplicates in the real-time PCR tests. The number of copies of the *Legionella* genome in the initial purified DNA solution was calculated by assuming an average molecular mass of 660 Da for one bp of double-stranded and using the following equation:

Thenumberofcopies=quantityofDNA(fg)/meanmassoftheL.pneumophilagenome

The genome of *L. pneumophila* means mass was calculated to be 3.72 fg from the mean size of the genome, which is assumed to be 3.4 Mb. This initial DNA solution was then divided into aliquots. An aliquot was serially diluted for each Light Cycler protocol to prepare six standard points containing 10^0^ to 10^5^. The standard contained 5.93 μL copies of DNA per 1000 μL solution. The bacterial DNA load per litre was calculated from the standard curve.

### HeLa cell culture and invasion assessment of isolates

HeLa cells (10^4^) were seeded in 5% of Dulbecco's modified Eagles medium in 96-well plates. Each plate was contained 10% heat-inactivated fetal bovine serum (Gibco) at 37°C in a moist air atmosphere. Bacteria were added to monolayer cells in each well at the multiplicity of infection (MOI) of 100. Then the plate was centrifuged for 5 min at 900×g and incubated for 1 hour at 37°C. Extracellular bacteria were killed by gentamicin (50 μg/mL) treatment for 1 hour. HeLa cells were washed twice with phosphate-buffered saline and lysed with cold distilled water [28].

Colony counts were obtained from plates containing 30–300 colonies. The number of CFU per gram of tissue is given by

cfu/ml=(no.ofcoloniesxdilutionfactor)/volumeofcultureplate formula.

The invasion percentage for each strain was calculated from the bacterial populations of the wells without gentamicin as follows:

%ofinvasion=[(#intracellularbacteria/mL)/(#bacteriaininoculum/mL)]×100

### Statistical analysis

SPSS 24 software was used to analyse demographic data (IBM, Armonk, NY, USA), and p ≤ 0.05 was considered statistically significant.

## Results

### Isolation and identification

One hundred water samples were collected from the IUMS various hospitals. Of these 100 samples, 48 cases (48%) were collected from showerheads, 44 cases (44%) from humidifier bottles, and eight cases (8%) from the bathwater.

**Fig. 1:**
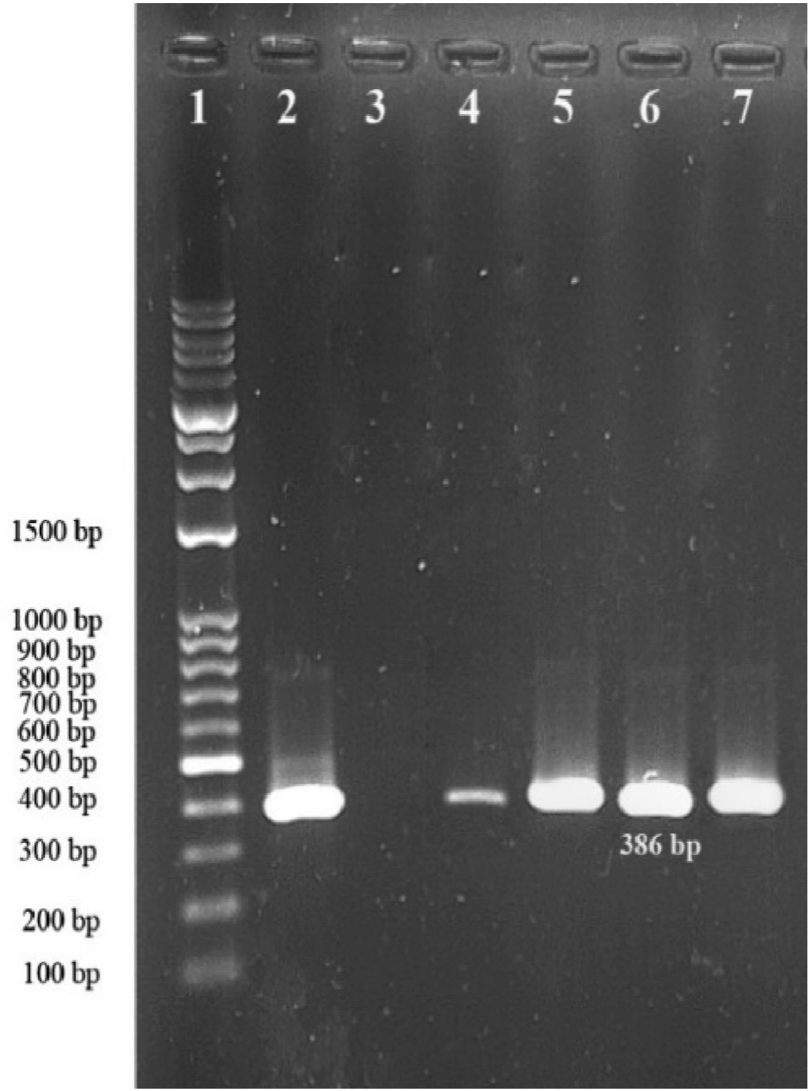
Electrophoresis of PCR product for 16S rRNA gene in samples. 1: 100bp Ladder/2: positive control: *L. pneumophila* ATCC33152 strain: 386 bp/3: negative control/4, 5, 6, 7: positive samples: 386 bp.

The samples were directly inoculated on the GVPC media. After incubation, the conventional culture method was used to isolate 12 cases (12%) from samples. These gram-negative isolates were identified based on colony morphology, growth on GVPC media, and lack of growth on standard media such as Blood agar and BCYE without L-Cys. In the conventional culture method, all 12 positive samples had less than 10^4^ CFU per litre, the top limit indicating a potential human health concern. The PCR approach revealed that 42 (42%) of the 100 samples were positive (Fig. 1), which the showerheads having the highest proportion (50%) and the bathwater samples having the lowest percentage (9.5%).

PCR-positive cases to positive culture cases were approximately 3.5, indicating that PCR was more sensitive than culture. The hospital with the highest percentage of PCR isolates was 12 cases (28.5%). The lowest percentage from another hospital was nothing (0%). Of all samples, 12 (12%) were positive by both culture and PCR methods, whereas 30 (30%) were positive only by PCR and had negative culture results.

### Quantification by qPCR method

**Fig. 2:**
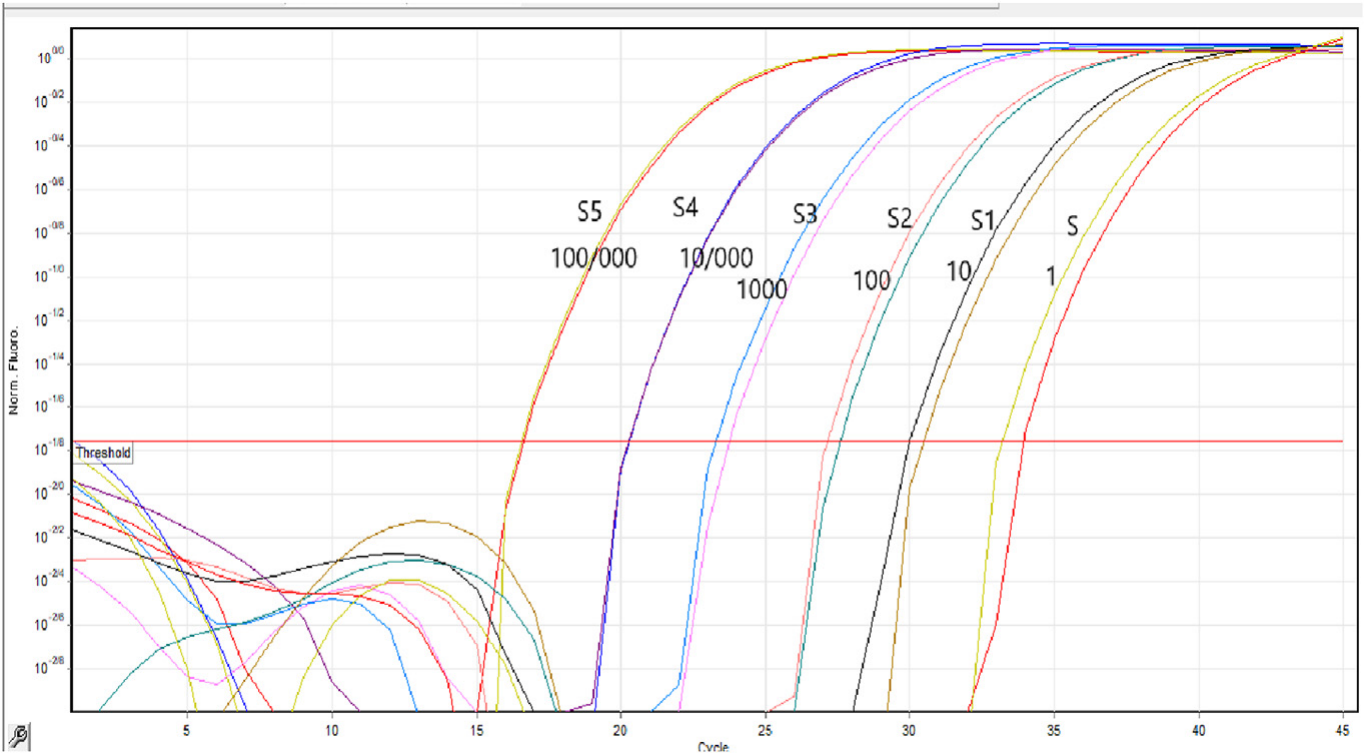
Quantitative PCR results using Cyber-green with specific primers (16S rRNA gene): For 16S rRNA, the detection and quantification limits (LOQ) were estimated to be 1GU/reaction (1000 GU/L).

**Fig. 3:**
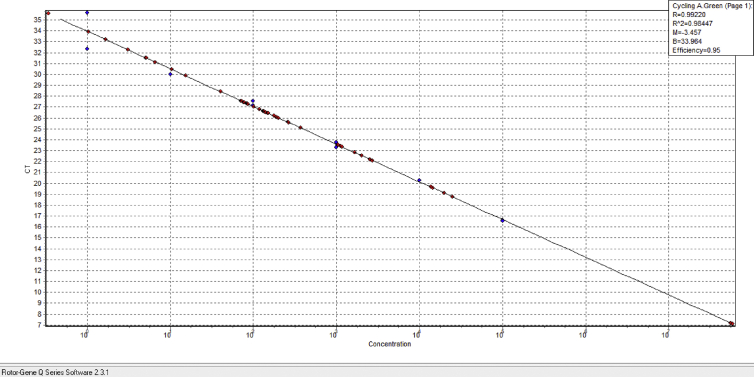
The qPCR results of water samples on the standard curve.

A linear correlation of 100 to 10^5^ copies per reaction mixture was seen after amplifying a 6-fold serial dilution of linearized *L. pneumophila* (ATCC 33152) genomic DNA samples, resulting in a 212-bp fragment with an R^2^ value of 0.99 and efficiency of 0.95. The detection and quantification limits (LOQ) were estimated to be 1 GU/reaction (1000 GU/L) for the 16S rRNA gene (Fig. 2). The values obtained from this method were computed based on the volume of water samples taken from various sources and the amount of DNA used in the qPCR process to determine the *Legionella* genome quantification per litre (Fig. 3). According to this method, of 42 PCR-positive water samples, 23 (54.76%) contained 10^4^ GU/L, 2 (4.76 percent) contained <10^4^ and about 10^3^ GU/L. Seventeen cases (40.47%) that were PCR positive were uncountable by the qPCR method (lower than 10^3^).

### Invasion assessment by HeLa cell culture

**Fig. 4:**
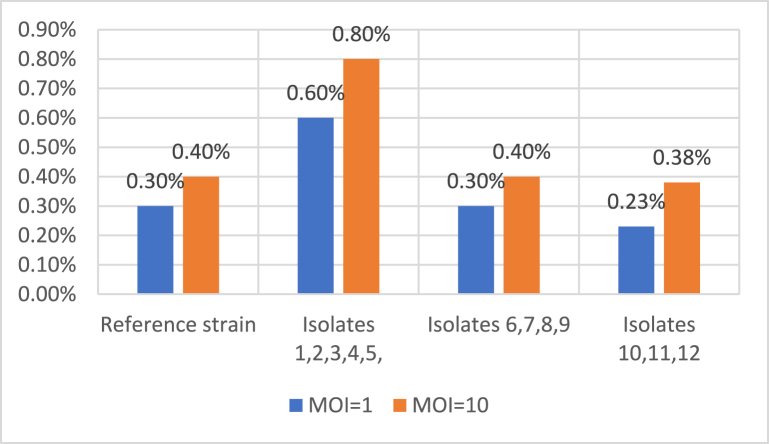
Invasion of HeLa cell by reference strain and isolates 1 hour after infected HeLa cells with *L. pneumophila* strains.

The HeLa cells were infected by 12 isolates. One hour after infection, the number of invaded *Legionella* was counted. Five isolates invaded HeLa cells more than twice as many times as the reference strain. Four isolates invaded at the same rate as the reference strains, whereas the rest invaded at a lower rate than the reference strain (Table 2; Fig. 4).

**Table 2:** Colony count of bacteria with MOI = 1 and MOI = 10

Isolates NO	MOI = 1	MOI = 10
Reference	3000	40 000
Isolates 1, 2, 3, 4, 5	6000	80 000
Isolates 6, 7, 8, 9	3000	40 000
Isolates 10, 11, 12	2300	38 000

## Discussion

According to the findings of our study, of 100 water samples collected from water sources of hospitals affiliated with IUMS, 12 (12%) were positive by culture, and 42 (42%) were positive by molecular PCR. *Legionella* spp. have been isolated directly from water sources as the gold standard. However, the culture method has disadvantages, such as the need for a specialized medium and supplementary elements for the organism's growth, the long incubation time, the growth of other bacterial species in similar conditions, and the inability to cultivate *Legionella* from some specimens. As identification and confirmation approaches, molecular techniques such as PCR and real-time PCR can be useful [17]. In the study by David R. Murdoch et al. in 2013 [32], the researchers examined standard methods for isolating and detecting *Legionella* bacteria. Of 114 samples tested over 4 years, 57 were positive by culture, and 99 were positive by PCR. The study's most notable finding was that the rate of *Legionella* detection by PCR was four times greater than expected culture results. After comparing the results of culture and PCR in our study, the rate of PCR to culture was found to be 3.5, which confirms the findings of earlier investigations.

In the study was conducted by Tabatabaei et al. in 2016 aiming isolation and identification of *Legionella* spp. from different aquatic sources in the south-west of Iran by molecular and culture methods, four of the 34 water samples taken were isolated using the culture method, whereas 14 were identified using the PCR method. The findings of this study underscored the need to control *Legionella* contamination in water sources and underlining the sensitivity and usefulness of the PCR approach in identifying *Legionella* bacteria in water samples [33].

Among other recent studies conducted in Iran by Moosavian et al. in 2019, isolation and identification of *Legionella* spp. in environmental water sources based on macrophage infectivity potentiator (mip) gene sequencing in south-west Iran. One hundred fourteen water samples were infected on the BCYE medium, with 20 cultured and subsequently processed using the PCR technique. According to the findings of this study, the presence of *Legionella* bacteria, particularly in hospital water supplies, can be dangerous; thus, continuous monitoring of hospital water sources can help minimize *Legionella* infections [31].

Water sources polluted with *Legionella* spp. according to estimates, at a rate of 10^2^–10^5^ CFU per litre of water, could be the source of occasional infections. Concurrently, polluted sources containing more than 10^5^ CFU per litre can spread the Legionnaires' epidemic [17]. As reported in prior investigations, the results demonstrated that qPCR was more sensitive than conventional culture for detecting *Legionella* [34,35]. In the present study, for 16S rRNA, the detection and quantification limits (LOQ) were estimated to be 1GU/reaction (1000 GU/L). Of 42 PCR-positive samples, 23 cases (54/76%) were with 10^4^ GU/L, two cases (4/76%) with <10^4^ GU/L, 17 cases (40/47%) were with <10^3^ GU/L. Based on previous studies, the findings we obtained can be used to evaluate and monitor the hospital water system at IUMS. In the study by s. Collins et al. in 2015, aiming to confirm the real-time PCR for detecting *Legionella* spp. and *L. pneumophila* serogroup 1 was compared with the culture-based detection method. There were 200 environmental samples collected. Culture and qPCR tests revealed that 38 of the samples tested positive for *Legionella* species. Both methods yielded negative results on 100 samples. Sixty-two samples were culture-negative but qPCR positive at the same time. As a result, qPCR can be used as a supplement to screening negative and positive samples more quickly [36]. In the study by Jonas Behets et al. in 2007, the number of genomic units counted was more significant than the number of CFUs found in water samples. This finding backs up previous findings, indicating that the culture method frequently underestimates the presence of *Legionella* in water samples. This study reported 100% specificity and 98% sensitivity for qPCR [34,37]. Also, in the study by Toplitsch et al. in 2021, to compare Updated Methods for *Legionella* Detection in Environmental Water Samples, 64 samples were examined for *Legionella* contamination. This bacterium was identified and counted using conventional culture and qPCR methods in this study. This study concluded that qPCR strongly recommended screening out *Legionella*-negative samples, especially for samples with a presumed high microbial burden before beginning labour-intensive culture methods [38]. Considering the preceding and the consistency of previous studies' results with our study of hospital water resources of IUMS, from which sampling was performed, in addition to being infected with *Legionella* bacteria, the allowable amount of this bacterium in water sources is close to the warning sign; and the qPCR method is a useful method as a supplementary of conventional culture for screening *Legionella* in water samples.

The isolates' invasion rates were compared to the standard strain in the final step of this study. The most important finding in this study was that the number of colonies increased as the MOI and cell incubation time increased, which also was observed in other studies. In the study by Lawrence A. Dreyfus [28] in 1989, The invasion of *L. pneumophila* virulent strain and its non-virulent and isogenic isolate in HeLa cells was compared. Although the non-virulent strain did not invade the cells in the same laboratory conditions, increasing MOI resulted in up to 100 bacterial penetration. According to the findings of this study, the number of colonies in the non-virulent strain in the MOI near 100 was equivalent to the number of colonies observed in the virulent strain of *L. pneumophila* (Lp1-vir) in the MOI 1. Even a short incubation time of 5–15 minutes for the virulent strain was sufficient for each MOI [28]. In another study conducted in 2013 by Masato Tachibana et al., 22 water samples were collected from various locations, and five cases (5%) were *L. pneumophila*. When the invasion of isolates was compared with the invasion of the reference strain, it was discovered that the isolates had a more severe invasion and intracellular growth than the reference strain [39]. As our study results showed, the invasion of HeLa cells was found to increase with increasing MOI.

## Conclusion

The study discovered that the spread of LD in hospitals was related to the hospital's water system. In a hospital population, there are always patients susceptible to infection and at high risk of *Legionella*. Transmit of this bacteria is commonly found in hospitalized patients' water. Given the importance of nosocomial infections in the medical community, establishing a hospital water monitoring system is the most effective way to treat, control, and prevent these infections, particularly *Legionella* infections. Because *Legionella* is a global health concern, reporting a false negative or failing to report a *Legionella* concentration is a serious risk. We can conclude that the findings of our study provide data and some insight into the possible detection of *Legionella* spp. in water sources in educational IUMS hospitals, which would help investigate any future outbreaks of LD in the hospital water system.

### Study limitations

*Legionella* is a fastidious organism that requires a high-quality and nutrient medium to grow. The hand preparation of a high-quality medium for this bacterium and its optimization to increase the possibility of isolating bacteria from collected water sources were among the limitations encountered in this study. Also, the slow growth of *Legionella* necessitates the elimination of competing for microbial flora through harsh methods such as heat and acid treatment, which are also thought to harm *Legionella* cultivability and may result in significant losses. Working with this bacterium was difficult because of its high tolerance to biocides, heat, and even acid and its ability to persist.

## Glossary

CAP: Community-acquired pneumonia
NAP: Nosocomial acquired pneumonia
BCYE: Buffered charcoal yeast extract
GVPC: Glycine, Vancomycin, Polymyxin, Cycloheximide
VBNC: Viable but non-culturable
PCR: Polymerase chain reaction
qPCR: Quantitative PCR
GU: Genome units
DMEM: Dulbecco's modified Eagles medium
IUMS: Iran university of medical sciences

## Authors’ contributions

S.B. contributed to conceptualization, methodology, investigation, resources, writing, reviewing, and editing the article, and visualization. S.M. contributed to conceptualization, methodology, validation, formal analysis, resources, writing the original article, visualization, supervision, project administration, and funding acquisition. F.M. contributed to project administration and writing the original article. D.D.-S. contributed to project administration and writing the original article. B.G.M. contributed to investigation and writing the original article.

## Transparency declaration

There is no conflict of interest declared.

## Ethical approval

The ethics committee approved this study of the Iran University of Medical Sciences (97-02-30-32879). Approval ID: IR.IUMS.REC.1397.010.
